# Notes from a London Clinic

**Published:** 1887-06

**Authors:** William B. Meany

**Affiliations:** London, England


### FOREIGN CORRESPONDENCE.

NOTES FROM A LONDON CLINIC.

LONDON, ENGLAND, April 15th, 1887.

*Special Correspondence of The Chicago Medical Journal and Examiner:*

I send you herewith some notes of cases treated by Sir Joseph Lister. In a case of nævas in a child four months old, in the operating theater of King's College hospital, he stated that the mother of the child said that at birth the nævus was a mere speck. In the short period since the child's birth it had grown to the size of three inches both in breadth and length. It was situated behind the angle of the jaw on the right side, covering the entire sterno-cleido-mastoid to within half an inch of the scapula. The extremely rapid growth of the tumor called, in the opinion of Sir Joseph, for surgical interference, although, as he stated, he could not feel certain that it could be successfully removed.

Further delay, however, if the nævus continued to increase in size as it had done, he said, would soon prove fatal. The patient being anæsthetized, the parts about the growth were cleanly shaven, and a towel wrung out of carbolized water, on which the instruments were placed, was at hand. The operator then made an incision into the healthy tissues about four lines beyond the margin of the nævus and encircling the entire growth. The incision reached to the fascia. The integument was picked up by a forceps and fingers, and the nævus gradually torn from its attachments, the knife being used to divide the tissues when their resistance proved too great for the fingers. The arteries were ligated as they were exposed, and then cut by scissors. When the growth had been entirely removed, there was left an opening so large that the anterior triangle and the greater portion of the posterior triangle of the neck were exposed.

All bleeding vessels were "picked up" with artery forceps until hemorrhage ceased, and each vessel ligated with silk-worm gut sutures; not more than two teaspoonsful of blood was lost. The wound was cleaned by sponges and carbolized water. Its edges were approximated by sutures of silver wire, introduced in sueh a manner as to cross each other in the shape of the letter X. This necessarily left an opening in the central part of the wound, and at each of its angles. Further approximation of the parts was secured by passing silk-worm gut sutures through those of the wire. Carbolized horsehair was introduced through such openings as remained, in order to secure drainage. Carbolized gauze and other carbolized materials were placed over the wound and the whole secured by a roller bandage, and an elastic linen band applied around the edges of the dressings.

An idea may be formed of the great extent of the wound after the removal of the nævus by stating that nineteen large silver-wire sutures were required to bring the integument into apposition. There were fifteen vessels ligated during the operation, silk-worm gut being used. The operator occupied one hour and fifteen minutes in his work. For some days previous to the operation the tumor had been injected with a solution of carbolic acid. After the growth had been removed, it was examined to see what changes, if any, had been effected in its structure by the carbolic acid. There was found simply a slight change of color in a few places, and a small degree of hardening or condensation of tissue. The nævus measured one and a half inches at its center. The operator stated that there was little danger of hemorrhage in removing a nævus if the incisions were made some distance from its margin into the healthy integument, tearing away by the aid of fingers and handle of knife where possible. The child was seen by the writer some hours after the operation, and appeared to have fully rallied from it.

Sir Joseph Lister brought to the notice of the writer two patients who had been operated upon seven days previously for cancer of the breast—the last operation being the second performed on one of the patients. The dressings were then removed for the first time. The wounds had healed by first intention. When sutures were removed they were found free from all trace of pus. The patients had no fever.

The "spray," as is now well known, has been discarded by Sir Joseph Lister for some time. The writer was present some twelve months or more ago at a clinic of Sir Joseph Lister, when he called attention to the absence of his "spray," stating that he was then sorry he had ever inflicted the "spray" upon the profession, or words to that effect. He continues to use, and places great reliance upon, his double chloride dressings.

WILLIAM B. MEANY, M.D.

